# A New Operation for the Radical Cure of Inguinal and Femoral Hernia

**Published:** 1895-03

**Authors:** Charles A. L. Reed

**Affiliations:** Cincinnati, Dean of the Faculty and Professor of Abdominal and Pelvic Surgery in the Cincinnati College of Medicine and Surgery (Medical Department of the University of Cincinnati); 487 W. Sixth Street


### Abstracts and Selections.

### A New Operation for the Radical Cure of Inguinal and Femoral Hernia.

BY CHARLES A. L. REED, A.M., M.D., CINCINNATI,
Dean of the Faculty and Professor of Abdominal and Pelvic Surgery in the Cincinnati College of Medicine and Surgery (Medical Department of the University of Cincinnati).

Hernia, when treated by surgical methods, is a safely curable condition. I place this declaration at the head of my paper for the reason that, by its position, I wish to make it emphatic. The necessity for this emphasis arises from the fact that many persons, not in this audience of select surgeons, but beyond this Association, in the medical profession and out of it, are victims of the opinion that nothing can be done for these cases of rupture. As a result, large numbers of persons endure inconveniences and pain arising from the protrusion or from a truss, or both, until they are overtaken with what too frequently proves to be a fatal strangulation. It has fallen to my lot, during the last twenty-years, to do a considerable number of operations for the relief of strangulated hernia. I can say, with entire candor, that no class of cases has ever impressed me so profoundly as have these unfortunate victims of neglect, for I assume that, in the light of the demonstrated results of surgical practice, cases of rupture that are not operated upon are essentially neglected. The agonizing pain, the intractable obstruction, the persistent vomiting, the active peritonitis, the fatal collapse, are too often the elements of the tragic picture that the surgeon is called upon to contemplate. It is precisely these conditions, in lesser or in greater degree, that sooner or later come to the majority of all victims of these forms of rupture. There is hardly a practitioner but that can recall cases in which an entirely reducible hernia had given no trouble for the twenty, thirty or more years since its occurrence, when all at once, and apparently without cause, it would slip past an even well-fitting truss, become strangulated, and utterly defy reduction except by surgical means, or, if these were withheld, end in death. It is a knowledge of exactly these facts, together with the pain and discomfort of a truss and an embarrassing sense of physical incapacity, that prompts almost every person who is ruptured to seek some means, not of palliation, but of cure. Advanced surgeons, such as Bassini, Macewen, Marcy and McBurney, have responded to this demand by devising operations that have reduced the mortality to a minimum and the primary recoveries to a maximum, but it can not be said that the medical profession, as a body, has appreciated this practice at its full value. On the contrary, these cases are either turned over to the truss vendor, or are permitted to fall into the hands of the surgical charlatan, whose only stock in trade is a squirt-gun and some tea made from white-oak bark.

The truss has done some good, and much harm. That it has been a valuable support to many people, that it has enabled many who were otherwise incapacitated to resume and maintain their avocations with a sort of comfort, and that the inflammation provoked by its pressure has closed the ring for a time in a few cases, comprises practically the sum total of its good results. On the other hand, that it has lured many persons into a false and even fatal sense of security, can not be denied. The most serious indictment, however, which can be brought against the truss, is that, probably in a majority of all cases in which it is employed, it only aggravates the real condition that it was intended to cure —a fact too frequently lost sight of simply because the pad, persistently applied, represses the hernial protrusion for the time being. The real damage is done by the truss in a two-fold way: The first consists in the fact that a "well-adjusted" pad must necessarily dilate the ring into which it is being continuously pushed by the elasticity of the spring; the second consists in the fact that the tissues around the ring waste, just as all tissues waste or atrophy under persistent pressure, and consequently, that the parts which it is so important to strengthen are really weakened in a very serious way by the truss.

The treatment by injecting irritating fluids under the skin, presumably into the ring, but really wherever they may lodge, needs only to be mentioned to be condemned. Although it was at one time subjected to careful trial by the medical profession, after its inauguration by Heaton, the verdict has been rendered against it. That it ever commanded sufficient recognition to be subjected to trial is due to the fact that it was vaunted as a method of practice before what now comprises modern surgery was elaborated. To-day the practice has no footing, except in the hands of persons outside the pale of the profession. The condemnation of the practice arises not only from its extreme danger and demonstrated inefficiency, but because it violates the most important axiom of surgery, namely, that all parts of the field of operation shall be under the inspection and control of the operator.

The operations which have been devised and generally practiced for the cure of hernia, whether done in connection with strangulation or as an elective procedure, have, to my mind, had a common defect, which, more than any one factor, has been responsible for the recurrences which mar the statistical tables of able operators. This defect consists in the fact that in all of the operations—Macewen's, McBurney's Marcy's, Bassini's—the attempt is made to do the operation through the canal itself, with the result that conditions within the pelvis which really cause the hernia, are left practically undisturbed. These conditions consist of an infundibular peritoneum and a serous track which, particularly in congenital cases of scrotal hernia, is left in primitive continuity, being only temporarily obstructed by inflammatory exudate. What is needed is to practically invert the infundibulum, close the internal ring, fix the cord in position of increased obliquity, and obliterate the canal.

An operation which I have devised, and which has proven primarily successful in my hands, and which meets the points I have just enumerated, is done as follows: The incision (in inguinal hernia) is made from a point two inches above Poupart's ligament, obliquely downward and inward, as nearly as possible coincident with the axis of the inguinal canal, to a point at the base of the scrotum. The dissection is then carried into both cavities. The protruding viscera is then reduced, and carefully inspected after being brought out above. The sac is then carefully dissected from its scrotal connections, and reversed by invagination. It is then opened by two incisions, one toward the pubis, the other toward the ilium, being thus converted into an anterior and a posterior flap. The cord is now dissected loose and caused to enter the canal, now denuded of its peritoneum, at its outer angle. The internal ring is now closed by several interrupted sutures, animal or pure silk, these sutures being applied beneath the peritoneal flaps formed by splitting the sac, care being taken that in the closure of the ring undue pressure shall not be brought to bear upon the cord. The posterior peritoneal flap is now excised, the stump being ligated should there be any demonstrated necessity for doing so. The anterior peritoneal flap is carried across the now obliterated internal ring, and is stitched by interrupted sutures to the posterior parietal peritoneum. This flap may be made broader by further dividing the peritoneum. The external ring is now closed by passing a number of sutures through its pillars external to the cord, which is now fixed in the internal (pubic) angle of the outlet of the canal. The incision into the abdomen is now closed by interrupted figure-of-eight sutures, the internal loop embracing the peritoneum, the aponeurosis of the transversalis and of both oblique muscles and the external loop, embracing the superficial fasciæ, fat and skin. These sutures should not be more than three-sixteenths of an inch apart. The incision into the scrotum may be closed in the ordinary way. Drainage should not be employed, except in the presence of marked oozing or obvious infection.

The reasons underlying these successive steps need but little elaboration. (1) The incision through the abdominal wall is important, because it places all of the conditions under the direct control of the operator. (2) The inversion and bisection of the sac furnishes two excellent instrumentalities for the further obliteration of the canal. (3) The complete enucleation of the cord from within the pelvis outward still further denudes the canal of its serous lining. (4) The suturing of the antero-posterior pillars of the internal ring strengthens the parieties at the most strategic point, and secures the occlusion of the canal by the deposit on it of firm cicatricial tissue. (5) The transplantation of the peritoneal flaps (*a*) destroys the infundibular form of the peritoneum and (*b*) completely protects the now closed ring from dilating by visceral pressure.

On general review of the operation I feel that it can be commended for the following reasons, viz.:

1. It is safe. There ought to be no deaths from it when done in uncomplicated cases under sanitary surroundings and by an operator skilled in abdominal surgery.

2. It causes but little loss of time. The surgical period is over within a week, although a longer time ought to be taken to permit the parts to become firm. A truss should not be worn after getting up.

3. It is comparatively painless. In the absence of internal ligatures compressing nerve trunks, it is gratifying to note the absence of all pain except the slight soreness of the superficial incision.

4. It is curative. The complete closure of the internal ring and the obliteration of the canal successfully prevent the recurrence of the hernia.

487 W. Sixth Street. —*Reprint.*

